# Preference-based measures of health-related quality of life in congenital mobility impairment: a systematic review of validity and responsiveness

**DOI:** 10.1186/s13561-020-00270-3

**Published:** 2020-04-21

**Authors:** Nathan Bray, Llinos Haf Spencer, Rhiannon Tudor Edwards

**Affiliations:** 1 School of Health Sciences, Fron Heulog, Bangor University, Gwynedd, LL57 2EF Wales, UK; 2 Centre for Health Economics and Medicines Evaluation, Ardudwy, Bangor University, Gwynedd, LL57 2PZ Wales, UK

**Keywords:** Disability, Mobility impairment, Quality of life, Health-related quality of life, Patient reported outcomes, Preference-based outcome measures, Utilities, QALYs

## Abstract

**Introduction:**

Mobility impairment is the leading cause of disability in the UK. Individuals with congenital mobility impairments have unique experiences of health, quality of life and adaptation. Preference-based outcomes measures are often used to help inform decisions about healthcare funding and prioritisation, however the applicability and accuracy of these measures in the context of congenital mobility impairment is unclear. Inaccurate outcome measures could potentially affect the care provided to these patient groups. The aim of this systematic review was to examine the performance of preference-based outcome measures for the measurement of utility values in various forms of congenital mobility impairment.

**Methods:**

Ten databases were searched, including Science Direct, CINAHL and PubMed. Screening of reference lists and hand-searching were also undertaken. Descriptive and narrative syntheses were conducted to combine and analyse the various findings. Results were grouped by condition. Outcome measure performance indicators were adapted from COSMIN guidance and were grouped into three broad categories: validity, responsiveness and reliability. Screening, data extraction and quality appraisal were carried out by two independent reviewers.

**Results:**

A total of 31 studies were considered eligible for inclusion in the systematic review. The vast majority of studies related to either cerebral palsy, spina bifida or childhood hydrocephalus. Other relevant conditions included muscular dystrophy, spinal muscular atrophy and congenital clubfoot. The most commonly used preference-based outcome measure was the HUI3. Reporting of performance properties predominantly centred around construct validity, through known group analyses and assessment of convergent validity between comparable measures and different types of respondents. A small number of studies assessed responsiveness, but assessment of reliability was not reported. Increased clinical severity appears to be associated with decreased utility outcomes in congenital mobility impairment, particularly in terms of gross motor function in cerebral palsy and lesion level in spina bifida. However, preference-based measures exhibit limited correlation with various other condition-specific and clinically relevant outcome measures.

**Conclusion:**

Preference-based measures exhibit important issues and discrepancies relating to validity and responsiveness in the context of congenital mobility impairment, thus care must be taken when utilising these measures in conditions associated with congenital mobility impairments.

## Introduction

### Mobility impairment and assistive mobility technology

Mobility impairment is the leading cause of disability in the UK, accounting for 52% of reported disabilities [1]. Mobility impairments arise from a vast array of different disabilities, conditions, injuries and illnesses. However, they can be classified broadly as either congenital (i.e. from birth) or acquired (i.e. occurring later in life). Whether a disability is present from birth or acquired later on in life significantly influences individual adaptation. For instance, individuals with congenital disabilities exhibit higher degrees of life satisfaction, self-identity and self-efficacy (related to their disability) than individuals who have had to adapt to acquired disability [2]. Adaptation to disability is influenced by self-concept and disability identity, which in turn are related to the onset of disability [2].

Common congenital conditions which can impact mobility include cerebral palsy (CP) and spina bifida (SB). CP refers to a number of conditions caused by damage to the parts of the brain which control movement, balance and posture, and can be caused either by abnormal brain development or trauma. CP is symptomized by varying degrees of permanent movement disorder, including poor coordination, muscle stiffness/weakness and involuntary movements. SB affects the development of the spine and spinal cord before birth, and can result in leg weakness and paralysis. There are three types of SB: myelomeningocele, meningocele and SB occulta.

Both congenital and acquired mobility impairments may necessitate the use of assistive technology to alleviate impairments. Assistive technology refers to a wide array of products and services which enhance functioning, participation and promote independence for people who have disabilities. The Medicines and Healthcare Products Regulatory Agency defines assistive technology as any device “intended to compensate for or alleviate an injury, handicap or illness or to replace a physical function” [3]. Assistive technology, such as wheelchairs, are an “essential component for inclusive sustainable development” [4], and can enhance the fundamental freedoms and equality of opportunity for people with mobility impairments and other disabilities. The United Nations (UN) states that access to appropriate and affordable assistive technology is a basic human right [5]; the UN Convention on the Rights of Persons with Disabilities has been ratified by 175 Member States, who are obligated to ensure that affordable assistive technology is available to all individuals in need. However, The World Health Organization (WHO) estimates that only 10% of people who need assistive technology have access to it [6], and there remain persistent challenges in the equitable provision of assistive technology, particularly in developing countries. One of the keys issues is in assessing the costs and benefits of different assistive technologies, and developing evidence-based approaches to provision which make best use of limited resources to maximise the outcomes of people with disabilities.

The National Health Service (NHS) in the UK spends almost £200million per year on wheelchairs alone [7], thus there is an imperative to ensure that assistive mobility technologies (AMTs) such as wheelchairs and other mobility-enhancing interventions are provided in an evidence-based manner, utilising evidence of cost-effectiveness to guide service commissioning.

### Economic evaluation and quality-adjusted life years

Methods of economic evaluation are now routinely embedded in the evaluation of health technologies, and used to estimate the cost of incremental benefits associated with new and alternative health interventions. Cost-utility analysis, specifically estimation of cost per quality-adjusted life years (QALYs), has become the predominant form of economic evaluation for new health technologies in the UK, in part due to the National Institute for Health and Care Excellence’s (NICE) advocacy for this approach [8]. The QALY framework has become increasingly influential in health policy as a theoretically universal and generic approach to measuring benefits via a single common outcome.

In order to calculate QALYs, health state utility values are needed. These values are most commonly derived from preference-based measures (PBMs) of health-related quality of life (HRQoL). HRQoL is a subjective and multi-dimensional construct defined as the perceived impact of health status on quality of life, including physical, psychological and social functioning. PBMs of HRQoL are used to assess the social desirability and utility values associated with different states of health.

As the descriptive systems and value sets of generic PBMs are usually derived from adult samples of the general population, a common criticism is that their genericity limits relevance and sensitivity in certain conditions [9]. Moreover, in health states where quality of life takes precedent over quantity of life (e.g. chronic illness, life-limiting conditions and disability), QALYs derived from generic PBMs can devalue the effectiveness of an intervention [10].

### Use of preference-based measures in the context of mobility impairment

The accuracy of a QALY estimate is subject to the sensitivity and applicability of the measurement tool used to generate the utility data. PBMs have been found to be inconsistent in both congenital and acquired mobility impairments [11–13], furthermore different PBMs produce significantly different results for AMT users [14, 15].

Previous research shows that patients with congenital mobility impairments do not necessarily consider mobility to have a major impact on their HRQoL when suitable adaptations (such as AMT) are available [16, 17]. However, general population PBM value sets heavily impact estimation of HRQoL when ability to walk is affected. As an example, using the NICE approved UK value set for the EuroQoL five-dimension (three level version) (EQ-5D-3 L), the lowest possible mobility level (‘confined to bed’) has a disutility of − 0.664, meaning that an individual who is unable to walk but is otherwise mobile using AMT can achieve a maximum utility value of 0.336 (0 = death; 1 = perfect health), even if they have no other HRQoL impacts. This raises the questions as to whether existing PBMs are a valid source of utility values in mobility impaired populations, particularly AMT users.

The validity of PBMs can be tested by comparing results across groups of patients and by comparing generic PBMs with condition-specific measures. For instance, the EQ-5D-3 L and the Health Assessment Questionnaire (HAQ; an outcome measure for rheumatoid arthritis) both measure health status in significantly different ways [18], suggesting that the EQ-5D-3 L is lacking consideration of important health impacts associated with rheumatoid arthritis. These issues are partly due to the insensitivity of the EQ-5D-3 L ‘mobility’ dimension to accurately assess the varied impacts of rheumatoid arthritis on mobility [19]. Similarly, the limited level choices on the EQ-5D-3 L have been found to cause some individuals with mobility impairments to choose levels which are more or less severe than their actual state, such as using ‘I am confined to bed’ to substitute being confined ‘to an electric wheelchair’ [20]. The updated five level version of the EQ-5D (EQ-5D-5 L) is unlikely to address this issue as the five level choices still focus on walking and do not take account of alternative methods of mobility.

Even simple generic measures of health status, such as the single question self-reported health (SRH) scale (i.e. “in general, would you say your health is excellent, very good, good, fair, or poor?”), exhibit only limited correlation with PBMs such as the Health Utilities Index (HUI) 3 and Assessment of Quality of Life (AQoL) in the context of SB [21] and CP [22]. Likewise, for individuals with spinal cord injuries, the wording of the 36-Item Short Form Health Survey (SF-36) (from which the Short-Form Six-Dimension (SF-6D) PBM is calculated) must be modified in order to maintain relevance [23].

Considering the potential issues of using generic PBMs in disability, and congenital mobility impairment specifically, it is apparent that there are a number of important considerations when using PBMs to evaluate AMT interventions and other mobility-enhancing interventions for people with congenital mobility impairments. The objective of this systematic review is therefore to examine the measurement properties of generic utility-based PBMs in various forms of congenital mobility impairment. All evidence reporting (or inferring) the validity, reliability and/or responsiveness of PBMs in conditions associated with congenital mobility impairment was collated and synthesised. Comparable condition-specific reviews of PBM performance have been conducted in mobility impairments such as rheumatoid arthritis [24], CP [25] and multiple sclerosis [26], however PBM performance has not been systematically summarised and collated across a range of congenital mobility impairments to date.

## Methods

This systematic review followed the University of York Centre for Reviews and Dissemination (CRD) principles for conducting searches and extracting data [27]. Internet reference database searching was the main strategy for gathering evidence. Databases included: Cochrane Collaboration Register and Library, Science Direct, CINAHL, ASSIA, PsychINFO, PubMed and Web of Science. Screening of reference lists, hand-searching and targeted searching via the CRD database (which covers the Database of Abstracts of Reviews of Effects (DARE), the NHS Economic Evaluation Database (NHS EED) and the Health Technology Association (HTA) database) were carried out in addition to the primary database searches. Due to limited translation resources, only studies written or translated into English or Welsh were eligible for inclusion. Search results were managed using the online bibliographic management software Refworks (for storage of titles from the systematic searches) and Mendeley (for referencing purposes). The systematic review protocol was registered on PROSPERO (CRD42018088932).

### Search terms

For the purpose of this review, mobility impairment was defined as any congenital (i.e. present from or shortly after birth) condition, impairment, disability or illness which causes significant restrictions to mobility for 12 months or longer, and which necessitates the use of AMT, surgery or rehabilitation to maintain, facilitate or substitute ambulation, or to reduce complications related to mobility impairment. Acquired mobility impairments (i.e. not present from birth) and short-term injuries, such as sprains or acute muscular injuries, were not included under this definition of mobility impairment.

Search terms included a mixture of MeSH (Medical Subject Heading) and non-MeSH words and phrases, divided into two groups: ‘population’ and ‘outcomes’ (see Table 1).

**Table 1:** Search terms and phrases

Population		Outcomes
Assisted mobility	Mobility scooter	15D
Assistive mobility	Mobility technolog*	AQoL
Brain damage*	Motor dis*	Assessment of Quality of Life
Brain injur*	Motorised scooter	Child health utilities
Buggy	Neurodisability	Child health utility
Caliper	Neurological dis*	CHU9D
Cane	Neuromotor dis*	CHU-9D
Cerebral palsy	Neuromuscular dis*	EQ 5D
Club foot	Orthoti*	EQ-5D
Clubfoot	Osteogenesis imperfecta	EuroQoL
Crutch*	Paraly*	Health utilities
Diplegi*	Paraplegi*	Health-utilities
Dysmelia	Physical disab*	HUI
Dystroph*	Physical impair*	HUI2
Electric chair	Physically disab*	HUI3
Electric powered indoor outdoor chair	Physically impaired	Preference based
Electric powered indoor/outdoor chair	Power chair	Preference-based
Electric scooter	Powered chair	QALY
Electrically powered indoor outdoor chair	Pushchair	Quality adjusted life year
Electrically powered indoor/outdoor chair	Quadriplegi*	Quality-adjusted life year
Electronically powered indoor outdoor chair	Rollator	Quality of well-being scale
Electronically powered indoor/outdoor chair	Scooter	QWB-SA
*encephal*	Spina bifida	Short Form Six Dimension
EPIOC	Spinal muscular atrophy	Short From 6 Dimension
Functional disab*	Talipes	SF6D
Handicap*	Tetraplegi*	SF-6D
Hemiplegi*	Walk aid	
Hydrocephalus	Walk-aid	
Knee scooter	Walker	
Knee walker	Walking aid	
Mobility aid	Walking frame	
Mobility device	Walking stick	
Mobility dis*	Walking-aid	
Mobility equipment	Wheelchair	
Mobility impair*		

^*^Indicates truncated words/phrases

In order to identify studies referring to interventions rather than patient groups (e.g. studies examining ‘wheelchair users’ more generally), the ‘population’ search terms also covered relevant AMTs and mobility-enhancing interventions. The ‘outcomes’ search terms covered relevant PBM keywords, including specific outcome measures (such as the various versions of the EQ-5D and HUI measures). An NHS posture and mobility service manager was consulted to refine the search terms. An example of a search string is shown in Table 2.

**Table 2:** Example of keyword search string

(“Assisted mobility” Or “Assistive mobility” Or “Brain damage*” Or “Brain injur*” Or Buggy Or Caliper Or Cane Or “Cerebral palsy” Or “Club foot” Or Clubfoot Or Crutch* Or Diplegi* Or Dysmelia Or Dystroph* Or “Electric chair” Or “Electric powered indoor outdoor chair” Or “Electric powered indoor/outdoor chair” Or “Electric scooter” Or “Electrically powered indoor outdoor chair” Or “Electrically powered indoor/outdoor chair” Or “Electronically powered indoor outdoor chair” Or “Electronically powered indoor/outdoor chair” Or encephal* Or EPIOC Or “Functional disab*” Or Handicap* Or Hemiplegi* Or Hydrocephalus Or “Knee scooter” Or “Knee walker” Or “Mobility aid” Or “Mobility device” Or “Mobility dis*” Or “Mobility equipment” Or “Mobility impair*” Or “Mobility scooter” Or “Mobility technolog*” Or “Motor dis*” Or “Motorised scooter” Or Neurodisability Or “Neurological dis*” Or “Neuromotor dis*” Or “Neuromuscular dis*” Or Orthoti* Or “Osteogenesis imperfect” Or Paraly* Or Paraplegi* Or “Physical disab*” Or “Physical impair*” Or “Physically disab*” Or “Physically impaired” Or “Power chair” Or “Powered chair” Or Pushchair Or Quadriplegi* Or Rollator Or Scooter Or “Spina bifida” Or “Spinal muscular atrophy” Or Talipes Or Tetraplegi* Or “Walk aid” Or “Walk-aid” Or Walker Or “Walking aid” Or “Walking frame” Or “Walking stick” Or “Walking-aid” Or Wheelchair) AND (15D OR AQoL OR “Assessment of Quality of Life” OR “Child health utilities” OR “Child health utility” OR CHU9D OR “CHU-9D” OR EQ. 5D OR “EQ-5D” OR EuroQoL OR “Health utilities” OR “Health-utilities” OR HUI OR HUI2 OR HUI3 OR “Preference-based” OR “Preference based” OR QALY OR “Quality adjusted life year” OR “Quality of well-being scale” OR “Quality-adjusted life year” OR “QWB-SA” OR “Short Form Six Dimension” OR “Short From 6 Dimension” OR SF6D OR “SF-6D”)	

### Study eligibility

Any study reporting the performance of PBMs of HRQoL in patient groups with congenital mobility impairments was eligible for inclusion. This included studies reporting proxy outcomes. There was no restriction on study type. Studies focussing solely on non-PBMs of HRQoL or acquired mobility impairments were excluded. Studies which included patient groups with varying degrees of disability/disease severity were considered for inclusion if the majority of patients had a congenital mobility impairment or if the data for patients with congenital mobility impairments was reported separately (i.e. sub-group analysis).

### Screening

Two researchers undertook each stage of the screening process. For the initial screening process, all identified studies were assessed for relevance based on their title and descriptor terms, the remaining studies were then assessed by their abstract. All studies considered relevant after the initial screening process were then obtained in full. Both reviewers screened each study independently. A third researcher was consulted when there was disagreement about the inclusion of a specific study.

### Quality appraisal

All relevant studies which met the initial inclusion criteria were critically appraised for methodological quality by two researchers. Quality appraisal methods were adapted from similar systematic reviews in other clinical areas [28–30]. Quality appraisal was not used to exclude studies, but to illustrate the overall quality of research conducted in this topic area. Quality appraisal focussed on six key areas:

- Whether tests of statistical significance were carried out
- Differences between interventions and/or patient groups (i.e. sub-group analysis)
- Clinical significance and relevance of results
- Reporting of missing data
- Response and completion rates
- Explicit reporting of inclusion/exclusion criteria

### Data extraction

Data extraction criteria included:

- Study characteristics: study type, country, number/composition of study groups, missing data
- Demographics: number of participants, age, gender, type/severity of mobility impairment
- Measures: Generic PBMs used, condition-specific measures used, other clinically relevant measures used
- Outcomes: Mean utility scores, mean utility scores for relevant sub-groups, statistical significance between groups
- Performance: Known group analyses, convergent validity (correlation between outcomes and/or respondent types), responsiveness, reliability, response/completion rates

### Analysis of the performance of preference-based measures

PBM performance indicators were adapted from COSMIN measurement property guidance for health-related patient-reported outcomes [31].

#### Assessment of validity

In this context validity refers to the extent to which a PBM can be considered to measure what it has been designed to measure (i.e. HRQoL), and whether it does so in a systematic manner. By establishing whether a specific PBM is sufficiently valid in a particular patient group, greater confidence can be placed in the generated data. We focussed predominantly on construct validity in this review.

Construct validity was assessed in a number of ways. Firstly, known-group analyses were used to assess whether specific PBMs were able to detect expected differences between different patient groups (i.e. variance due to severity of illness). Secondly, convergent validity was determined by examining correlation between comparable outcomes, for instance between PBMs and condition-specific measures with comparable constructs. Finally, convergent validity was further examined by looking at correlation between respondents types (i.e. self-reported and proxy utility outcomes). In the interest of uniformity, the strength of correlations was defined as absent (*r* < 0.20), weak (*r* = 0.20 to 0.35), moderate (*r* = 0.35 to 0.50) and strong (*r* ≥ 0.50) [32].

#### Assessment of reliability

Reliability refers to the replicability of results. Reliability is commonly assessed by examining test-retest results and inter-rater reliability of PBMs in defined unchanging patient groups.

#### Assessment of responsiveness

Responsiveness refers to the extent to which a measure can identify changes in health status [28]. A responsive measure should be able to detect clinically significant changes in health outcomes over time [29]. Responsiveness was determined by examining the relationship between outcomes derived from PBMs and other relevant measures, before and after an intervention.

### Evidence synthesis

Descriptive synthesis of search results was conducted [27] and presented in tabulated form. Narrative synthesis was undertaken to develop a structured narrative of results; extracted results were grouped by type of mobility impairment and category of PBM performance.

## Results

**Fig. 1:**
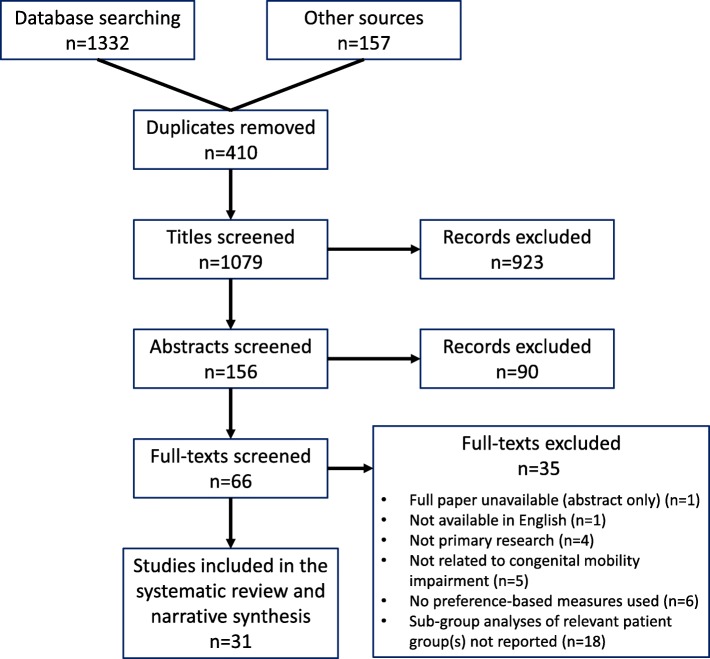
PRISMA flowchart for search outcomes and screening process

See Fig. 1 for search outcomes and the screening process flowchart. Searches were conducted from March to May 2018. In total 1489 study articles were identified: 1332 from the bibliographic searches and 157 articles from other sources (i.e. CRD database, screening of reference lists and hand-searching), of which 410 duplicates were removed. After screening of titles and abstracts, 66 studies were identified as potentially eligible. Following full review of full-texts, 35 studies were excluded for a variety of reasons (see Fig. 1). In total 31 studies were identified as relevant and eligible for inclusion in the systematic review. Quality appraisal outcomes are presented in Table 3.

**Table 3:** Quality appraisal outcomes

Study reference (author, year)	Tests of statistical significance conducted	Sub-group analyses conducted	Clinical implications discussed	Proportions of missing/incorrect data reported	Response and/or completion rates reported	Inclusion and/or exclusion criteria explicitly stated
Bartlett et al. (2010) [33]	**Y**	**Y**	**Y**	**Y**	**X**	**Y**
Bray et al. (2017) [15]	**Y**	**Y**	**Y**	**Y**	**Y**	**Y**
Burstrom et al. (2014) [11]	**Y**	**Y**	**X**	**Y**	**Y**	**X**
Cavazza et al. 2016 [34]	**X**	**X**	**X**	**X**	**X**	**X**
Christensen et al. (2016) [35]	**Y**	**Y**	**Y**	**X**	**Y**	**X**
Findlay et al. (2015) [36]	**Y**	**Y**	**Y**	**Y**	**Y**	**X**
Hendriksz etl al (2014) [37].	**Y**	**Y**	**Y**	**Y**	**X**	**Y**
Karmur and Kulkarni (2018) [38]	**Y**	**X**	**Y**	**Y**	**X**	**X**
Kennes et al. (2002) [39]	**Y**	**Y**	**Y**	**Y**	**X**	**Y**
Kulkarni et al. (2004) [40]	**Y**	**X**	**Y**	**X**	**Y**	**Y**
Kulkarni (2006) [41]	**Y**	**Y**	**X**	**Y**	**X**	**Y**
Kulkarni et al. (2008) [42]	**Y**	**X**	**Y**	**Y**	**Y**	**X**
Kulkarni et al. (2008) [43]	**Y**	**Y**	**Y**	**X**	**Y**	**Y**
Landfeldt et al. (2016) [44]	**Y**	**X**	**Y**	**X**	**Y**	**X**
Lindquist et al. (2014) [45]	**Y**	**Y**	**Y**	**X**	**X**	**Y**
Livingston and Rosenbaum (2008) [46]	**Y**	**X**	**Y**	**Y**	**X**	**X**
Lopez-Bastida et al. (2017) [47]	**X**	**X**	**Y**	**Y**	**X**	**X**
Morrow et al. (2011) [48].	**Y**	**Y**	**Y**	**X**	**Y**	**Y**
Penner et al. (2013) [49]	**Y**	**Y**	**Y**	**X**	**Y**	**X**
Perez Sousa et al. (2017) [50]	**Y**	**Y**	**Y**	**Y**	**Y**	**Y**
Petrou and Kupec (2009) [51]	**Y**	**Y**	**Y**	**Y**	**X**	**Y**
Rocque et al. (2015) [52]	**Y**	**Y**	**Y**	**X**	**X**	**Y**
Rosenbaum et al. (2007) [53]	**Y**	**Y**	**Y**	**Y**	**X**	**Y**
Sims-Williams et al. (2016) [54]	**Y**	**Y**	**Y**	**Y**	**X**	**Y**
Slaman et al. (2015) [55]	**Y**	**X**	**Y**	**X**	**X**	**Y**
Tilford et al. (2005) [56]	**Y**	**X**	**Y**	**X**	**Y**	**X**
Usuba et al. (2014) [57]	**Y**	**Y**	**Y**	**Y**	**Y**	**Y**
Vitale et al. (2001) [58]	**Y**	**Y**	**Y**	**X**	**X**	**Y**
Wallander et al. (2009) [59].	**Y**	**X**	**Y**	**X**	**Y**	**Y**
Young et al. (2010) [22].	**Y**	**Y**	**Y**	**Y**	**Y**	**Y**
Young et al. (2013) [21]	**Y**	**Y**	**Y**	**X**	**Y**	**Y**

### Study and patient characteristics

Study and participant characteristics are presented in Table 4. Most of the studies (22 of 31) were cross-sectional studies [15, 21, 22, 34, 36–38, 40–45, 47–52, 54, 58, 59], six were prospective cohort studies [33, 35, 39, 46, 53, 57], two were case-control studies [11, 56] and one was a randomised controlled trial [55]. The selected studies were conducted in a range of different countries; most were (*n* = 15) from Canada [21, 22, 33, 35, 36, 38–43, 46, 49, 53, 57]. The sample sizes of relevant study groups ranged from 13 [15] to 770 [44].

**Table 4:** Study characteristics and participant demographics for included studies

Study reference	Aims and objectives	Country	Study type	Condition(s) of interest	Clinical and diagnostic information (% of sample)	Sample size	Age (mean ± SD; range) and gender (% of sample)
Bartlett et al. (2010) [33]	To explore possible reasons for the observed decline in gross motor capacity of adolescents with cerebral palsy in GMFCS levels III, IV and V	Canada	Prospective cohort	Adolescents with cerebral palsy	*GMFCS level* GMFCS III: 38% GMFCS IV: 35% GMFCS V: 27% *Cerebral palsy sub-type* Diplegia: 24% Hemiplegia: 1% Tetraplegia: 71%	*n* = 135	Mean 14 yrs. (±2.4; range 11–17) 44% female / 56% male
Bray et al. (2017) [15]	To compare how children with mobility impairments and their parents (by proxy) report HRQoL using standard outcome measures	UK	Cross-sectional	Children and adolescents with impaired mobility (relevant conditions: cerebral palsy, hemiplegia, muscular dystrophy)	*Mixed diagnoses* Cerebral palsy: 85% Hemiplegia/stroke: 8% Muscular dystrophy: 8%	*n* = 13	Range 6-18 yrs. 39% female / 62% male
Burstrom et al. (2014) [11]	To test the feasibility and validity of the EQ-5D-Y in a Swedish patient sample of children and adolescents with functional motor, orthopaedic and medical disabilities and to compare the results with a general population sample	Sweden	Case-control	Children and adolescents with functional motor, orthopaedic and medical disabilities (relevant conditions: artrogryposis multiple congenital, myelomeningocele, cerebral palsy, orthopaedic lower limb deformities, achondroplasia)	*Mixed diagnoses* Artrogryposis multiple congenital: 14% Myelomeningocele: 17% Cerebral palsy: 20% Orthopaedic lower limb deformities: 7% Achondroplasia: 6%	*n* = 71 case group *n* = 407 control group	*Case group* Mean 12 yrs. (±3.1; range 7–17) 61% female / 39% male *Control group* Mean 13 yrs. (±2.7; range 8–16) 49% female / 51% male
Cavazza et al. (2016) [34]	To determine the economic burden from a societal perspective and the HRQoL of patients with Duchenne muscular dystrophy, in Europe	Multinational (Bulgaria, France, Germany, Hungary, Italy, Spain, Sweden, UK)	Cross-sectional	Adolescents and adults with Duchenne muscular dystrophy	Not stated	*n* = 268	Mean age varied by country, from 11 yrs. (±5.6) in Sweden to 23 yrs. (±15.8) in Bulgaria. 70% of sample were children (range 2–17) 7% female / 93% male
Christensen et al. (2016) [35]	To identify factors associated with a change in pain over time in children with cerebral palsy	Canada	Prospective cohort	Children and adolescents with cerebral palsy	*GMFCS level* GMFCS I: 21% GMFCS II: 11% GMFCS III: 24% GMFCS IV: 22% GMFCS V: 22%	*n* = 148	Mean 8 yrs. (range 3–19) 30% female / 70% male
Findlay et al. (2015) [36]	To explore whether HRQoL can be predicted by pain, age, GMFCS level, and sex in children with cerebral palsy and whether different pain aetiologies have varying effects on HRQoL	Canada	Cross-sectional	Children with cerebral palsy	*GMFCS level* GMFCS I: 26% GMFCS II: 16% GMFCS III: 23% GMFCS IV: 18% GMFCS V: 17% *Cerebral palsy sub-type* Unilateral: 19% Bilateral spastic: 75% Dyskinetic: 4% Other (ataxic and hypotonic): 2%	*n* = 248	Mean 10 yrs. (±4.3) 37% female / 63% male
Hendriksz et al. (2014) [37]	To assess the global burden of disease among patients with Morquio A syndrome, including impact on mobility/wheelchair use, HRQoL, pain and fatigue and the interaction between these factors	Multinational (Brazil, Colombia, Germany, Spain, Turkey, UK)	Cross-sectional	Children and adults with Morquio A syndrome	*Comorbidities* Bone deformity: 75% full sample Abnormal gait: 96% adult group / 75% child group	*n* = 36 child group *n* = 27 adult group	*Child group* Range 5-17 yrs. (47% aged 10–14) 44% female / 56% male *Adult group* Range 18-40 yrs. (52% aged 18–24) 44% female / 56% male
Karmur and Kulkarni (2018) [38]	To understand the quality of life of patients with myelomeningocele and shunted hydrocephalus	Canada	Cross-sectional	Children and adolescents with spina bifida (myelomeningocele) and shunted hydrocephalus	Not stated	*n* = 131	Mean 12 yrs. (±3.7) 51% female / 49% male
Kennes et al. (2002) [39]	To describe the health status of pre-adolescent children with cerebral palsy, and to determine the strength of correlations between the severity of gross motor functional impairment and other aspects of functional health status (sensory, intellectual, emotional etc.)	Canada	Prospective cohort	Children with cerebral palsy	*GMFCS level* GMFCS I: 28% GMFCS II: 12% GMFCS III: 20% GMFCS IV: 20% GMFCS V: 21%	*n* = 408	Mean 8 yrs. (±1.9; range 5–15) 46% female / 54% male
Kulkarni et al. (2004) [40]	To develop and test the psychometric properties of the Hydrocephalus Outcome Questionnaire (HOQ), as a measure of health status in clinical research projects of paediatric hydrocephalus	Canada	Cross-sectional	Children with hydrocephalus	*Hydrocephalus aetiology* Congenital/aqueductal stenosis: 36% Myelomeningocele: 13% Other: 51%	*n* = 90	Mean 10 yrs. (±3.5) Gender distribution not stated
Kulkarni et al. (2006) [41]	To compare three separate methods for establishing interpretability for the HOQ, and to calculate the conversion of numerical HOQ scores into utility scores obtained from HUI2	Canada	Cross-sectional	Children with hydrocephalus	*Hydrocephalus aetiology* Congenital/aqueductal stenosis: 33% Myelomeningocele: 15% Intraventricular haemorrhage: 13% Other: 40%	*n* = 79	Mean 10 yrs. (±3.5) Gender distribution not stated
Kulkarni et al. (2008) [42]	To study the factors associated with HRQoL in Canadian children with hydrocephalus, using a comprehensive model of determinants of child health, including socioeconomic factors	Canada	Cross-sectional	Children with hydrocephalus	*Hydrocephalus aetiology* Myelomeningocele: 33% Intraventricular haemorrhage of prematurity: 9% Aqueductal stenosis: 10% Post-infection: 4% Posterior fossa cyst: 5% Not stated: 39%	*n* = 340	Mean 11 yrs. (±3.6) Gender distribution not stated
Kulkarni et al. (2008) [43]	To investigate the feasibility and scientific properties of a child-completed version of the HOQ (cHOQ)	Canada	Cross-sectional	Children with hydrocephalus	*Hydrocephalus aetiology* Myelomeningocele: 34% Intraventricular haemorrhage of prematurity: 11% Aqueductal stenosis: 10% Post-infection: 4% Congenital communicating: 3% Intracranial cyst: 8% Other: 30%	*n* = 273	Mean 14 yrs. (±2.6) 47% female / 54% male
Landfeldt et al. (2016) [44]	To estimate HRQoL in patients with Duchenne muscular dystrophy	Multinational (Germany, Italy, UK, USA)	Cross-sectional	Children and adolescents with Duchenne muscular dystrophy	*Ambulatory status* Early ambulatory (5-7 yrs): 20% Late ambulatory (8-11 yrs): 33% Early non-ambulatory (12-15 yrs): 20% Late non-ambulatory (≥16 yrs): 27%	*n* = 770	Mean 14 yrs. (±7.0) 100% male
Lindquist et al. (2014) [45]	To analyse quality of life in a very long-term follow-up of now adult individuals, treated for hydrocephalus (without spina bifida) during their first year of life	Sweden	Cross-sectional	Adults who experienced hydrocephalus in infancy	31% of study group diagnosed with cerebral palsy and/or epilepsy; hydrocephalus aetiologies not reported	*n* = 29 study group *n* = 1613 control group	*Study group* Mean 34 yrs. (range 30–41) 38% female / 62% male *Control group* Matched age and gender to case group
Livingston and Rosenbaum (2008) [46]	To assess the stability of measurement of quality of life and HRQoL over the course of 1 year among adolescents with cerebral palsy	Canada	Prospective cohort	Adolescents with cerebral palsy	*GMFCS level* GMFCS I: 30% GMFCS II: 16% GMFCS III: 15% GMFCS IV: 25% GMFCS V: 15%	*n* = 185	Mean 16 yrs. (±1.75; range 13–20) 47% female / 54% male
Lopez-Bastida et al. (2017) [47]	To determine the economic burden and health-related quality of life of patients with spinal muscular atrophy and their caregivers in Spain	Spain	Cross-sectional	Children with spinal muscular atrophy	*Spinal muscular atrophy type* Type I: 10% Type II: 74% Type III: 16%	*n* = 81	Mean 7 yrs. (±5.47) 58% female / 42% male
Morrow et al. (2011) [48]	To evaluate differences between children’s, parents’ and doctors’ perceptions of health states and HRQoL in children with chronic illness and explore factors which explain these differences	Australia	Cross-sectional	Children with chronic conditions (relevant condition: cerebral palsy)	All participants in cerebral palsy sub-group were categorised as GMFCS level V	*Cerebral palsy sub-group* *n* = 1 child-parent pair *n* = 1 child-doctor pair *n* = 11 parent-doctor pairs	*Cerebral palsy sub-group* 36% aged > 12 yrs. Gender distribution not reported
Penner et al. (2013) [49]	To determine the impact of pain on activities and to identify the common physician-identified causes of pain in children and youth aged 3 to 19 years across all levels of severity of cerebral palsy	Canada	Cross-sectional	Children and adolescents with cerebral palsy	*GMFCS level* GMFCS I: 24% GMFCS II: 13% GMFCS III: 21% GMFCS IV: 19% GMFCS V: 23%	*n* = 252	Mean 9 yrs. (±4.2; range 3–19) 36% female / 64% male
Perez Sousa et al. (2017) [50]	To analyse the level of agreement between children with cerebral palsy and their parents, using the EQ-5D-Y questionnaire and its proxy version	Spain	Cross-sectional	Children and adolescents with cerebral palsy	*Functional classification* Grade 1 (without activity limitation): 66% Grade 2 (mild or moderate activity limitation): 34%	*n* = 62	Mean 10 yrs. (±2.3; range 6–17) 44% female / 56% male
Petrou and Kupek (2009) [51]	To augment previous catalogues of preference-based HRQoL weights by estimating preference-based HUI3 multiattribute utility scores associated with a wide range of childhood conditions	UK	Cross-sectional	Children with childhood conditions (relevant conditions: microcephaly, cerebral palsy, spinal muscular atrophy, muscular dystrophy, spina bifida)	Not stated	*Relevant sub-groups* Microcephaly *n* = 40 Cerebral palsy *n* = 178 Muscular dystrophy or spinal muscular atrophy *n* = 45 Spina bifida *n* = 42	*Relevant sub-groups* Microcephaly: mean 11 yrs. Cerebral palsy: mean 11 yrs. Muscular dystrophy or spinal muscular atrophy: mean 12 yrs. Spina bifida: mean 13 yrs. Gender distribution per sub-group not reported
Rocque et al. (2015) [52]	To characterise the quality of life of paediatric patients with spina bifida, and to analyse factors that influence HRQoL and aid in the determination of whether a correlation exists between various disease and/or personal characteristics and HRQoL scores	USA	Cross-sectional	Children and adolescents with spina bifida	*Underlying diagnosis* Myelomeningocele: 79% Lipomyelomeningocele: 16% Meningocele: 3% Filum terminale-related pathology: 2% Sacral agenesis: > 1%	*n* = 159	Mean 12 yrs. (range 5–20) 57% female / 43% male
Rosenbaum et al. (2007) [53]	To report self- and proxy-assessed quality of life along with parental accounts of HRQoL of a cohort of adolescents with cerebral palsy participating in a longitudinal study charting mobility and self-care through the adolescent years	Canada	Prospective cohort	Adolescents with cerebral palsy	*GMFCS level* GMFCS I: 30% GMFCS II: 16% GMFCS III: 14% GMFCS IV: 25% GMFCS V: 16%	*n* = 203	Mean 16 yrs. (±1.75; range 13–20) 45% female / 55% male
Sims-Williams et al. (2017) [54]	To ascertain the quality of life of surviving children with spina bifida and to determine whether this was influenced by mobility, urinary continence, hydrocephalus, sex, family size and school attendance	Uganda	Cross-sectional	Children with spina bifida	45% of sample had comorbid hydrocephalus *Walking ability* Unable to walk: 47% Walk with sticks/crutches: 14% Walk unaided: 39%	*n* = 66 (63 of which completed HUI3)	Range 10-14 yrs. 44% female / 56% male
Slaman et al. (2015) [55]	To evaluate the cost-utility of a lifestyle interventions among adolescents and young adults with cerebral palsy	The Netherlands	Randomised controlled trial (single-blind)	Adolescents and young adults with cerebral palsy	Intervention group GMFCS level GMFCS I: 61% GMFCS II: 32% GMFCS III: 7% GMFCS IV: 0% Control group GMFCS level GMFCS I: 55% GMFCS II: 31% GMFCS III: 10% GMFCS IV: 4%	*n* = 20 intervention group *n* = 20 control group	*Intervention group* Mean 20 yrs. (±3.0) 57% female / 43% male *Control group* Mean 20 yrs. (±3.0) 48% female / 52% male
Tilford et al. (2005) [56]	To provide information on the preference scores of children with spina bifida aperta and to measure the impact of caring for a child with spina bifida consistent with economic evaluations	USA	Case-control	Children with spina bifida	*Case group lesion level* Sacral: 42% Lower lumbar: 34% Thoracic: 25%	*n* = 80 case group *n* = 30 general population control group	*Case group* Mean 9 yrs. (±4.6) 61% female / 39% male *General population control group* Mean 7 yrs. (±4.0) 55% female / 45% male
Usuba et al. (2014) [57]	To explore the magnitude and timing of changes in gross motor function and HRQoL among persons with cerebral palsy over an 8-year period, with specific interest in comparing those who made the transition to adult services	Canada	Prospective cohort	Adolescents and adults with cerebral palsy	*GMFCS level (full sample)* GMFCS I: 22% GMFCS II: 13% GMFCS III: 13% GMFCS IV: 22% GMFCS V: 30%	*n* = 31 ‘younger adults’ group *n* = 23 ‘older adults’ group	*‘Younger adults’ group* Mean 15 yrs. (range 13–17) *‘Older adults’ group* Mean 26 yrs. (range 23–32) *Full sample* 46% female / 54% male
Vitale et al. (2001) [58]	To examine whether the SF-36 and EQ-5D would be useful for evaluating quality of life in adolescents with orthopaedic conditions	USA	Cross-sectional	Adolescents with orthopaedic problems (relevant condition: cerebral palsy)	Not stated	*n* = 14 cerebral palsy sub-group	Cerebral palsy sub-group age data not reported (full sample: mean 14 yrs. [range 10–18]) Gender distribution not reported
Wallander et al. (2009) [59]	To review a group of patients over 60 years of age who had been treated for congenital talipes equinus varus (CTEV) in infancy, using generic instruments for the assessment of quality of life in general and a specific foot and ankle instrument for assessment of function	Sweden	Cross-sectional	Adults treated for CTEV in infancy	*Clubfoot laterality* Unilateral: 54% Bilateral: 46%	*n* = 83	Mean 64 yrs. (range 62–67) 24% female / 76% male
Young et al. (2010) [22]	To describe the health and quality of life outcomes of youth and young adults with cerebral palsy, and to explore the impact of 3 factors (cerebral palsy severity, age and sex) on quality of life outcomes	Canada	Cross-sectional	Adolescents and young adults with cerebral palsy	*‘Youth’ group GMFCS level* GMFCS I: 22% GMFCS II: 12% GMFCS III: 18% GMFCS IV: 25% GMFCS V: 22% *‘Adult’ group GMFCS level* GMFCS I: 23% GMFCS II: 14% GMFCS III: 19% GMFCS IV: 25% GMFCS V: 20%	*n* = 129 ‘youth’ group *n* = 70 ‘adult’ group	*‘Youth’ group* Mean 15 yrs. (±1.36) 45% female / 55% male *‘Adult’ group* Mean 26 yrs. (±2.63) 40% female / 60% male
Young et al. (2013) [21]	To describe the health and HRQoL outcomes of youths and young adults with spina bifida	Canada	Cross-sectional	Adolescents and young adults with spina bifida	*‘Youth’ group lesion level*Thoracic: 25% High-lumbar: 18% Low-lumbar: 30% Sacral: 28% *‘Adult’ group lesion level* Thoracic: 15% High-lumbar: 23% Low-lumbar: 31% Sacral: 15% Unknown: 15%	*n* = 40 ‘youth’ group *n* = 13 ‘adult’ group	*‘Youth’ group*Mean 16 yrs. (±1.3; range 13–17)65% female / 35% male *‘Adult’ group* Mean 26 yrs. (±3.10; range 23–32) 77% female / 23% male

Fourteen studies included data relating to CP [22, 33, 35, 36, 39, 46, 48–51, 53, 55, 57, 58], six included data relating to SB [21, 38, 51, 52, 54, 56] and five included data relating to childhood hydrocephalus [40–43, 45]. Data for a range of other relevant conditions were also found in either single studies or from extractable sub-groups, these included muscular dystrophy [34, 44], spinal muscular atrophy [47], Morquio A syndrome [37], congenital clubfoot [59] and microcephaly [51]. Three studies focused on multiple conditions where sub-group data could not be examined separately [11, 15, 51].

The vast majority of studies (23 of 31) included only child/adolescent participants [11, 15, 33, 35, 36, 38–44, 46–54, 56, 58]. Of the remaining studies, six included children/adolescents and adults [21, 22, 34, 37, 55, 57] and two included only adults [45, 59]. PBM reporting was predominantly from proxies (13 of 23 studies); eleven studies included both self-reported and proxy data [15, 21, 22, 36, 43, 48–50, 53, 54, 57] and seven studies included only self-reported PBM data [11, 34, 37, 45, 55, 58, 59].

In terms of use of PBMs, 22 of the studies used a version of the HUI [15, 21, 22, 33, 35, 36, 38–44, 46, 48, 49, 51–54, 56, 57], eight used a version of the EQ-5D [11, 15, 34, 37, 47, 50, 58, 59], three used a version of AQoL instrument [21, 22, 57], one used the 15D [45] and one used the SF-6D [55].

### Narrative synthesis

Utility outcomes are presented in Table 5 and PBM performance outcomes are presented in Table 6. The narrative synthesis is categorised by type of mobility impairment and PBM performance indicator. No studies reported PBM reliability outcomes, therefore reliability results have not been presented.

**Table 5:** Summary of utility outcomes for included studies

Study reference	Condition(s) of interest	Respondent type	PBM (s)	Other relevant outcome measures	Overall utility scores (mean ± SD)	Sub-group utility scores (mean ± SD)
Bartlett et al. (2010) [33]	Adolescents with cerebral palsy	Proxy (parent)	HUI3	Gross Motor Function Measure 66 Items (GMFM-66) Spinal Alignment and Range of Motion Measure (SAROMM)	NA Overall utility scores not reported, only vision, cognition and pain dimensions used - ambulation, hearing, speech, dexterity and emotion dimensions all excluded due to conceptual overlap with other measures	NA
Bray et al. (2017) [15]	Children and adolescents with impaired mobility (relevant conditions: cerebral palsy, hemiplegia, muscular dystrophy)	Self-reported and proxy (parent); matched-pairs	HUI2, HUI3 and EQ-5D-Y	Visual analogue scale (VAS)	*Child self-reported* HUI2: 0.53 (±0.07) HUI3: 0.22 (±0.09) EQ-5D-Y: 0.24 (±0.30) *Parent proxy* HUI2: 0.49 (±0.09) HUI3: 0.16 (±0.10) EQ-5D-Y: 0.01 (±0.14)	NA
Burstrom et al. (2014) [11]	Children and adolescents with functional motor, orthopaedic and medical disabilities (relevant conditions: artrogryposis multiple congenital, myelomeningocele, cerebral palsy, orthopaedic lower limb deformities, achondroplasia)	Self-reported	EQ-5D-Y	VAS KIDSCREEN-27 KIDSCREEN-10 Self-rated health (SRH) Life satisfaction ladder (LSL)	NA Overall utility scores not reported, only dimension scores reported	NA
Cavazza et al. (2016) [34]	Adolescents and adults with Duchenne muscular dystrophy	Self-reported	EQ-5D-Y	VAS Barthel Index	Mean 0.24 Varied by country, ranging from − 0.71 (±0.41) in Sweden to 0.66 (±0.08) in Bulgaria	NA
Christensen et al. (2016) [35]	Children and adolescents with cerebral palsy	Proxy (caregiver)	HUI3	Wong-Baker FACES Pain Rating Scale	NA Overall utility scores not reported, only HUI3 pain dimension measured	NA
Findlay et al. (2015) [36]	Children with cerebral palsy	Self-reported and proxy (caregiver); proportion of proxy data not reported	HUI3	DISABKIDS Chronic Generic module (DCGM-37) DISABKIDS Smiley measure (DSM) Wong-Baker FACES Pain Rating Scale	NA Overall utility scores not reported, only HUI3 pain dimension measured	NA
Hendriksz et al. (2014) [37]	Children and adults with Morquio A syndrome	Self-reported	EQ-5D-5 L	Brief Pain Inventory Short Form (BPI-SF) Adolescent Pediatric Pain Tool (APPT)	NA Overall utility scores not reported	*Child group: utility score by wheelchair use frequency* No use: 0.534 Occasional use: 0.664 Full-time use: − 0.180 *Adult group: utility score by wheelchair use frequency* No use: 0.846 Occasional use: 0.582 Full-time use: 0.057
Karmur and Kulkarni (2018) [38]	Children and adolescents with spina bifida (myelomeningocele) and shunted hydrocephalus	Proxy (caregiver)	HUI2 and HUI3 (version used to calculate utility scores not stated)	Hydrocephalus Outcome Questionnaire (HOQ)	0.51 (±0.28)	NA
Kennes et al. (2002) [39]	Children with cerebral palsy	Proxy (caregiver)	HUI3	Functional Independence Measure for Children (WeeFIM) Strength and Difficulties Questionnaire (SDQ) Wide Range Achievement Test (WRAT)	NA Overall utility scores not reported, only dimension scores reported	NA
Kulkarni et al. (2004) [40]	Children with hydrocephalus	Proxy (parent)	HUI2	HOQ	NA Overall utility scores not reported	NA
Kulkarni et al. (2006) [41]	Children with hydrocephalus	Proxy (parent)	HUI2	HOQ	NA Overall utility scores not reported	NA
Kulkarni et al. (2008) [42]	Children with hydrocephalus	Proxy (caregiver)	HUI3	HOQ	0.58 (±0.32)	NA
Kulkarni et al. (2008) [43]	Children with hydrocephalus	Self-reported and proxy (caregiver); proxy-reported HUI3 and HOQ, self-reported cHOQ	HUI3	Child-completed version of the HOQ (cHOQ)	0.71 (±0.27)	NA
Landfeldt et al. (2016) [44]	Children and adolescents with Duchenne muscular dystrophy	Proxy (caregiver); although patients included in data collection, only proxy utility data reported	HUI (version not stated)	Pediatric Quality of Life Inventory (PedsQL) neuromuscular module 3.0	NA Overall utility scores not reported	*Utility score by ambulatory status* Early ambulatory: 0.75 Late non-ambulatory: 0.15
Lindquist et al. (2014) [45]	Adults who experienced hydrocephalus in infancy	Self-reported	15D	NA	Study group: 0.92	Control group: 0.95
Livingston and Rosenbaum (2008) [46]	Adolescents with cerebral palsy	Proxy (parent)	HUI3	Quality of Life Instrument for People with Developmental Disabilities (QOL Instrument)	NA Overall utility scores not reported	NA
Lopez-Bastida et al. (2017) [47]	Children with spinal muscular atrophy	Proxy (caregiver)	EQ-5D-3 L	VAS Barthel Index	0.16 (±0.44)	*Utility score by spinal muscular atrophy type* Type II spinal muscular atrophy sub-group: − 0.01 (±0.35) Scores for Type I and III not reported
Morrow et al. (2011) [48]	Children with chronic conditions (relevant condition: cerebral palsy)	Self-reported and proxy (parent and doctor); matched groups	HUI2 and HUI3	NA	NA Overall utility scores not reported for relevant sub-group	NA
Penner et al. (2013) [49]	Children and adolescents with cerebral palsy	Self-reported and proxy (parent and physician); proxy-reported HUI3 pain score, self-reported Wong-Baker FACES Pain Scale	HUI3	Wong-Baker FACES Pain Rating Scale	NA Overall utility scores not reported, only HUI3 pain dimension measured	NA
Perez Sousa et al. (2017) [50]	Children and adolescents with cerebral palsy	Self-reported and proxy (mother and father); matched groups	EQ-5D-Y	VAS	NA Overall utility scores not reported, only dimension scores reported	NA
Petrou and Kupek (2009) [51]	Children with childhood conditions (relevant conditions: microcephaly, cerebral palsy, spinal muscular atrophy, muscular dystrophy, spina bifida)	Proxy (caregiver)	HUI3	NA	*Mean utility by condition* Microcephaly: 0.141 Cerebral palsy: 0.276 Muscular dystrophy or spinal muscular atrophy: 0.386 Spina bifida: 0.452	NA
Rocque et al. (2015) [52]	Children and adolescents with spina bifida	Self-reported and proxy (caregiver); predominantly proxy (11% self-reported)	HUI3	NA	NA Overall utility scores not reported	*Utility score by type of spina bifida* Myelomeningocele: 0.51 Closed spinal dysraphism: 0.77 *Utility score by treatment history* No history of shunt or CM-II decompression: 0.74 Shunt but no CM-II decompression: 0.49 Shunt and CM-II decompression: 0.29
Rosenbaum et al. (2007) [53]	Adolescents with cerebral palsy	Self-reported and proxy (parent); proxy-reported HUI3, 34% self-reported QOL Instrument	HUI3	QOL Instrument	0.42 (±0.41)	*Utility score by GMFCS level* GMFCS I: 0.84 (±0.20) GMFCS II: 0.50 (±0.31) GMFCS III: 0.39 (±0.31) GMFCS IV: 0.16 (±0.26) GMFCS V: − 0.08 (±0.23)
Sims-Williams et al. (2017) [54]	Children with spina bifida	Self-reported and proxy (caregiver); matched pairs	HUI3	VAS	*Child self-reported* 0.58 (95% CI 0.49–0.66) *Caregiver proxy* 0.55 (95% CI 0.47–0.63)	NA
Slaman et al. (2015) [55]	Adolescents and young adults with cerebral palsy	Self-reported	SF-6D	36-Item Short Form Survey (SF-36) - SF-6D utility outcomes derived from SF-36	*Baseline* Control: 0.74 (±0.12) Intervention: 0.75 (±0.10) *6 months post intervention* Control: 0.77 (±0.12) Intervention: 0.80 (±0.03)	NA
Tilford et al. (2005) [56]	Children with spina bifida	Proxy (caregiver)	HUI2	Quality of Well-Being scale (QWB)	Case group: 0.55 (±0.24)	*Utility by case group lesion level* Sacral lesion: 0.61 (±0.26) Lower lumbar lesion: 0.54 (±0.19) Thoracic lesion: 0.45 (±0.25) General population control group: 0.93 (±0.11)
Usuba et al. (2014) [57]	Adolescents and adults with cerebral palsy	Self-reported and proxy (undefined); predominantly proxy (40% self-reported)	HUI3 and AQoL	SRH	*Baseline (both groups)* HUI3: 0.29 (±0.39) AQoL: 0.35 (±0.33) *8-year follow-up (both groups)* HUI3: 0.29 (±0.38) AQoL: 0.35 (±0.32)	NA
Vitale et al. (2001) [58]	Adolescents with orthopaedic problems (relevant condition: cerebral palsy)	Self-reported	EQ-5D (version not stated)	SF-36	Cerebral palsy sub-group: 0.922	NA
Wallander et al. (2009) [59]	Adults treated for CTEV in infancy	Self-reported	EQ-5D (version not stated)	SF-36 VAS American Academy of Orthopaedic Surgeons Foot and Ankle Questionnaire	NA Overall utility scores not reported	NA
Young et al. (2010) [22]	Adolescents and young adults with cerebral palsy	Self-reported and proxy (caregiver); predominantly self-reported (45% proxy)	HUI3 and AQoL	SRH Health Assessment Questionnaire (HAQ)	Combined age groups HUI3: 0.30 (±0.42) AQoL: 0.28 (±0.33)	*Utility score by age group (HUI3 / AQoL)* ‘Youth’ group: 0.30 (±0.43) / 0.28 (±0.34) ‘Adult’ group: 0.31 (±0.40) / 0.28 (±0.314) *Youth group: utility score by GMFCS level (HUI3 / AQoL)* GMFCS I: 0.67 (±0.32) / 0.58 (±0.31) GMFCS II: 0.59 (±0.35) / 0.53 (±0.34) GMFCS III: 0.43 (±0.39) / 0.31 (±0.32) GMFCS IV: 0.08 (±0.25) / 0.06 (±0.12) GMFCS V: − 0.13 (±0.19) / 0.01 (±0.07) *Adult group: utility score by GMFCS level (HUI3 / AQoL)*GMFCS I: 0.64 (±0.30) / 0.52 (±0.32) GMFCS II: 0.50 (±0.39) / 0.33 (±0.24) GMFCS III: 0.53 (±0.27) / 0.39 (±0.27) GMFCS IV: 0.06 (±0.21) / 0.10 (±0.20) GMFCS V: − 0.14 (±0.20) / 0.02 (±0.06)
Young et al. (2013) [21]	Adolescents and young adults with spina bifida	Self-reported and proxy (caregiver); predominantly self-reported (15% proxy)	HUI3 and AQoL	SRHHAQ	Combined age groups HUI3: 0.52 (±0.28) AQoL: 0.34 (±0.24)	*Utility score by age group (HUI3 / AQoL)* ‘Youth’ group: 0.58 (±0.27) / 0.37 (±0.26) ‘Adult’ group: 0.36 (±0.27) / 0.25 (±0.17) *Utility score by lesion level (HUI3 / AQoL)* Thoracic: 0.29 (±0.14) / 0.22 (±0.14) High-lumbar: 0.44 (±0.31) / 0.28 (±0.23) Low-lumbar: 0.63 (±0.23) / 0.39 (±0.21) Sacral: 0.76 (±0.20) / 0.51 (±0.33) Unknown: 0.22 (±0.02) / 0.09 (±0.04)

**Table 6:** Summary of PBM performance for included studies

Study reference	Condition(s) of interest	Known-group analyses	Construct validity: comparing outcomes	Construct validity: comparing PBMs	Construct validity: comparing respondents	Responsiveness
Bartlett et al. (2010) [33]	Adolescents with cerebral palsy	HUI3 vision, cognition and pain dimensions steadily declined as GMFCS level increased, statistical significance not reported.	Examining correlation coefficients, there was no indication that the HUI3 vision (*r =* 0.01; *p* = 0.92), cognition (*r =* − 0.05; *p* = 0.59) or pain dimensions (*r =* 0.16; *p* = 0.07) were determinants of motor capacity, as measured using the GMFM-66.	NA	NA	Individuals with a GMFCS level of V exhibited the largest mean decreases in HUI3 dimension levels over time, ranging from − 0.2 (±1.2) for the HUI3 vision dimension to − 0.3 (±1.3) for the HUI3 cognition and pain dimensions.
Bray et al. (2017) [15]	Children and adolescents with impaired mobility (relevant conditions: cerebral palsy, hemiplegia, muscular dystrophy)	NA	NA	Large variance between mean utility scores derived from different PBMs, ranging from 0.24 (EQ-5D-Y) to 0.53 (HUI2) for child self-reported utility, and from 0.01 (EQ-5D-Y) to 0.49 (HUI2) for parent-reported proxy utility.	A significant respondent type effect was found, with mean child self-reported utility scores significantly (*p* ≤ 0.021) higher than equivalent proxies on all PBMs. Significant strong correlations were found between utility scores for children/parent proxies using all measures: EQ-5D-Y (*r =* 0.67; *p* = 0.026), HUI2 (*r =* 0.73; *p* = 0.005) and HUI3 (*r =* 0.84; *p* < 0.001). Using Bland-Altman plots, sufficient agreement between utility scores for children/parent proxies was found for the HUI2 (CL = 0.22) and HUI3 (CL = 0.22). EQ-5D-Y exhibited clinically important discrepancies between child and parent proxy responses (CL = 1.04).	NA
Burstrom et al. (2014) [11]	Children and adolescents with functional motor, orthopaedic and medical disabilities (relevant conditions: artrogryposis multiple congenital, myelomeningocele, cerebral palsy, orthopaedic lower limb deformities, achondroplasia)	Statistically significant (*p* ≤ 0.001) differences between case and control groups on all dimensions. Mean dimension scores not reported, proportions of patients choosing each dimension level indicate case group reported more problems on all dimensions than the control group: 83% of case group reported some/a lot of problems on any dimension, compared to 37% of control group; likewise 21% of case group reported extreme problems on any dimension, compared to 2% of control group.	Strong significant correlation was found between the EQ-5D-Y anxiety/depression dimension and the KIDSCREEN-27 psychological well-being dimension (*r =* − 0.51; *p* = 0.001); KIDSCREEN-27 physical well-being dimension (*r =* − 0.53; *p* = 0.001); and life satisfaction ladder (LSL) (*r =* − 0.54; *p* < 0.001). Moderate correlation also found between this dimension and the KIDSCREEN-10 HRQoL index score (*r =* − 0.50; *p* = 0.001) and self-reported health (SRH) (0.42; *p* = 0.007). The self-care EQ-5D-Y dimension exhibited moderate correlation with the KIDSCREEN-27 physical wellbeing dimension (*r =* − 0.37; *p* = 0.028) and the usual activities EQ-5D-Y dimension was moderately correlated with the KIDSCREEN-27 psychological wellbeing dimension (*r =* − 0.35; *p* = 0.027). All other correlations were weak or absent.	NA	NA	NA
Cavazza et al. (2016) [34]	Adolescents and adults with Duchenne muscular dystrophy	NA	NA	NA	NA	NA
Christensen et al. (2016) [35]	Children and adolescents with cerebral palsy	NA	Using multivariate linear regression, a significant association was found between the HUI3 pain dimension score at baseline and GMFCS level (b = − 0.11, β = − 0.15; *p* < 0.036; 95% CI − 0.21 to − 0.01): higher pain score at baseline was associated with greater improvement in pain status in GMFCS level I compared to level V.	NA	NA	Using one-way ANOVA analysis, a significant association was found between physician primary pain aetiology and change in HUI3 pain status (*p* = 0.001): children with musculoskeletal pain at baseline showed significant improvements (mean change 0.55; 95% CI 0.053 to 1.05) compared to children without pain at baseline (mean change − 0.39; 95% CI − 0.62 to − 0.15) (p = 0.006). No other associations were reported. HUI3 pain dimension scores did not change significantly over time: median score of 2 (out of 5) at both visits. However, 55% of children changed pain status over time: 34% worsening, 21% improving.
Findlay et al. (2015) [36]	Children with cerebral palsy	NA	NA	NA	NA	NA
Hendriksz et al. (2014) [37]	Children and adults with Morquio A syndrome	A significant effect of wheelchair use on utility outcomes was reported; significant differences reported between: adult non-wheelchair users and occasional wheelchair users (*p* = 0.0115); adult occasional wheelchair users and full-time wheelchair users (*p* = 0.0007); child occasional wheelchair users and full-time wheelchair users (p = 0.0018); and child non-wheelchair users and full-time wheelchair users (*p* = 0.0018). Children who occasionally used a wheelchair had a higher mean utility score on average than non-wheelchair users, although both groups had higher average utility scores than full-time wheelchair users.	NA	NA	NA	NA
Karmur and Kulkarni (2018) [38]	Children and adolescents with spina bifida (myelomeningocele) and shunted hydrocephalus	Using multivariate regression analysis, anatomical level of myelomeningocele had a significant effect on utility score (*p* = 0.01): lower anatomical level of myelomeningocele was associated with a higher utility score.	NA	NA	NA	NA
Kennes et al. (2002) [39]	Children with cerebral palsy	NA	Using Kendall’s tau-b test of association, the HUI3 dimension most associated with GMFCS level was ambulation (tau-b = 0.82; *p* < 0.01): higher GMFCS level was associated with increased mobility impairment. Moderate correlations (ranging from tau-b = 0.36 to 0.58; *p* < 0.01) were found between GMFCS level and the vision, speech and dexterity dimensions. Overall patterns were similar to the ambulation dimension, but correlations were weaker. Hearing (tau-b = 0.16; *p* = 0.04) and cognition (tau-b = 0.27; *p* < 0.01) dimensions had statistically significant but low association with GMFCS level. The emotion (tau-b = 0.03; *p* = 0.24) and pain (tau-b = 0.07; *p* = 0.37) dimensions were not significantly associated with GMFCS level.	NA	NA	NA
Kulkarni et al. (2004) [40]	Children with hydrocephalus	NA	Strong correlation was found between utility score and the HOQ overall health (*r =* 0.81), physical health (*r =* 0.88), social-emotional (*r =* 0.56) and cognitive (*r =* 0.57) scores.	NA	NA	NA
Kulkarni et al. (2006) [41]	Children with hydrocephalus	NA	Correlation between utility score and HOQ overall health score was high (*r =* 0.81), a scatterplot demonstrated a strong linear relationship. Simple and complex linear regression models both accounted for a large proportion of HUI2 variability (adjusted R^2^ = 0.66 and 0.80 respectively).	NA	NA	NA
Kulkarni et al. (2008) [42]	Children with hydrocephalus	NA	Mean utility score (0.58 ± 0.63) close to cHOQ mean scores for overall health (0.65 ± 0.20), Physical health (0.66 ± 0.25), Cognitive health (0.55 ± 0.28) and Social-emotional health (0.71 ± 0.19). Statistical significance not reported.	NA	NA	NA
Kulkarni et al. (2008) [43]	Children with hydrocephalus	NA	Significant correlation was found between the cHOQ overall health score (self-reported) and utility score (proxy-reported) (*r =* 0.60; *p* < 0.001).	NA	NA	NA
Landfeldt et al. (2016) [44]	Children and adolescents with Duchenne muscular dystrophy	Ambulatory class was significantly associated with proxy-reported utility scores (*p* < 0.001), decreasing from early ambulatory class (mean 0.75) to late non-ambulatory class (mean 0.15). Caregiver assessments of health and mental status were significantly associated with utility score (*p* < 0.001).	NA	NA	NA	NA
Lindquist et al. (2014) [45]	Adults who experienced hydrocephalus in infancy	The study group had significantly (*p* ≤ 0.004) lower dimension scores compared to the control group in vision, eating, usual activities, mental function dimensions. The most reported problems were associated with the neuroimpairment subgroup (mean utility score 0.87 compared to 0.94 in the no neuroimpairment subgroup). The subgroup without neuroimpairment were not significantly different to the control in terms of mean utility score (0.94 and 0.95 respectively, p value not reported).	NA	NA	NA	NA
Livingston and Rosenbaum (2008) [46]	Adolescents with cerebral palsy	NA	Disattenuated correlation coefficients between utility scores and QOL Instrument scores demonstrated weak to moderate correlation for the being (*r =* 0.48); belonging (*r =* 0.35); becoming (*r =* 0.29) and overall quality of life (*r =* 0.35) scales. The two measure shared up to 23% variance.	NA	NA	Generalizability coefficients were calculated to assess variability of scores over time. Dimensions with greater stability (i.e. larger G scores) had less variability between individuals over time. The ambulation dimension (G = 0.94) and overall utility (G = 0.91) were found to be highly stable; while the speech (G = 0.87), vision (G = 0.87); dexterity (G = 0.82), cognition (G = 0.81), and hearing (G = 0.72) dimensions were moderately stable. The pain (G = 0.48) and emotion (G = 0.24) dimensions were found to have low stability.
Lopez-Bastida et al. (2017) [47]	Children with spinal muscular atrophy	Children with Type II spinal muscular atrophy were found to have a lower average utility score (− 0.012) than the combined average of children with all types of spinal muscular atrophy (0.158). Statistical significance was not reported.	NA	NA	NA	NA
Morrow et al. (2011) [48]	Children with chronic conditions (relevant condition: cerebral palsy)	NA	NA	NA	Agreement between respondents was assessed using Cohen’s kappa coefficient. Moderate agreement was found between parents of children with cerebral palsy and doctors for the HUI2 dimensions of sensation (63.6% agreement; Kappa 0.41), cognition (70%; Kappa 0.56) and self-care (100%; Kappa 1). Only the HUI3 ambulation dimension (63.6%; Kappa 0.46) demonstrated moderate agreement. All other dimensions exhibited slight or fair agreement	NA
Penner et al. (2013) [49]	Children and adolescents with cerebral palsy		A significant negative correlation was found between the HUI3 pain dimension and GMFCS level (*r =* 0.36; *p* < 0.001).	NA	Good correlation was found between child self-reported pain (Wong-Baker scale) and proxy reported pain (HUI3 pain dimension) (Goodman and Kruskall’s y = 0.57; *p* < 0.001). Using the HUI3 pain dimension, physicians identified pain in 4.4% of cases where parent proxies did not identify pain. In 17.4% of cases, parent proxies identified pain when physicians did not. Statistical significance not reported.	NA
Perez Sousa et al. (2017) [50]	Children and adolescents with cerebral palsy	NA	NA	NA	Child/father agreement was poor for all EQ-5D-Y dimensions (Kappa range 0.016–0.067; non-significant). Child/mother agreement between dimensions was mostly poor (Kappa range 0.057–0.389; non-significant), however for the mobility dimensions agreement was good (Kappa 0.713; *p* = 0.000) and for the usual activities dimension agreement was moderate (Kappa 0.436; *p* = 0.000). Mothers and father both tended to report fewer problems (by proxy) than the child.	NA
Petrou and Kupek (2009) [51]	Children with childhood conditions (relevant conditions: microcephaly, cerebral palsy, spinal muscular atrophy, muscular dystrophy, spina bifida)	Microcephaly: Adjusted disutility from perfect health was estimated to be − 0.820 (95% CI − 0.670 to − 0.970). Adjusted disutility from childhood norms was estimated to be − 0.745 (95% CI − 0.598 to − 0.899). Cerebral palsy: Adjusted disutility from perfect health was estimated to be − 0.726 (95% CI − 0.607 to − 0.846). Adjusted disutility from childhood norms was estimated to be − 0.652 (95% CI − 0.536 to − 0.775). Muscular dystrophy and spinal muscular atrophy: Adjusted disutility from perfect health was estimated to be − 0.616 (95% CI − 0.471 to − 0.761). Adjusted disutility from childhood norms was estimated to be − 0.541 (95% CI − 0.400 to − 0.690). Spina bifida: Adjusted disutility from perfect health was estimated to be − 0.552 (95% CI − 0.404 to − 0.701). Adjusted disutility from childhood norms was estimated to be − 0.478 (95% CI − 0.333 to − 0.630).	NA	NA	NA	NA
Rocque et al. (2015) [52]	Children and adolescents with spina bifida	Diagnosis and type of spina bifida had a significant effect on overall utility and certain dimension scores. Overall utility was found to be significantly lower (*p* < 0.001) in children and adolescents with myelomeningocele compared to individuals with closed dysraphism. The HUI3 ambulation dimension was significantly lower (*p* < 0.001) in individuals with open myelomeningocele compared to individuals with closed neural tube defects. The HUI3 cognition dimension was significantly lower (*p* = 0.039) in individuals with open myelomeningocele compared to individuals with closed neural tube defects Utility and ambulation scores were significantly associated with bowel/bladder continence (*p* < 0.05). Those with bowel continence had higher average utility scores. Utility scores were significantly lower for patients with a history of receiving shunt and/or CM-II decompression interventions (*p* < 0.005). The dimensions of cognition (*p* = 0.17) and ambulation (p = 0.002) were found to be significantly lower for individuals with a history of shunting, while the dimensions of speech (*p* = 0.01) and cognition (*p* = 0.005) were significantly lower for individuals with a history of CM-II decompression. The results remained significant when controlled for age; shunt status accounted for 10.5% of variability in overall utility scores. Shunt revision status weakly correlated with utility score (*r =* − 0.197; *p* = 0.048) and dimension scores for vision, speech, ambulation (coefficients not given; p values ranged from 0.01 to 0.05).	NA	NA	NA	NA
Rosenbaum et al. (2007) [53]	Adolescents with cerebral palsy	GMFCS level was found to have a significant impact on utility score (*p* < 0.01); average utility scores decreased steadily as GMFCS level increased. Post-hoc Bonferoni correction confirmed significant differences in mean overall utility scores between all GMFCS levels (p values not reported), except between levels II and III (*p* = 0.82). HUI3 dimension levels were also significantly associated with GMFCS level for all dimensions (*p* < 0.05).	A strong negative correlation was found between utility score and GMFCS level (*r =* − 0.81; p value not reported) Adolescents’ ability to self-report using the QOL Instrument was significantly associated (*p* < 0.01) with parent-reported HUI3 ratings of speech (tau-b = 0.52); cognition (tau-b = 0.50); dexterity (tau-b = 0.35); ambulation (tau-b = 0.31); vision (tau-b = 0.22) and hearing (tau-b = 0.19). Utility score was significantly (p value not reported) but weakly correlated with scores on the QOL Instrument for Being (*r =* 0.37), Belonging (*r =* 0.17), Becoming (*r =* 0.20), and Overall quality of life (*r =* 0.28). Utility scores explained between 2.9% (Belonging) and 14% (Being) of variance in QOL Instrument scores.	NA	NA	NA
Sims-Williams et al. (2016) [54]	Children with spina bifida		Moderate correlation was found between child-reported (*r =* 0.49) and proxy-reported (*r =* 0.38) utility scores and child-reported VAS - the authors describe this as a ‘poor’ correlation, p values not reported.	NA	Child self-reported and caregiver proxy utility scores were highly correlated (*r =* 0.85; p value not reported).	NA
Slaman et al. (2015) [55]	Adolescents and young adults with cerebral palsy	NA	NA	NA	NA	No significant difference between the control and intervention groups at the end of the trial (*p* = 0.42). QALYs gained equated to 0.78 for the control group, and 0.79 for the intervention group; the incremental difference of 0.013 was not significant (*p* = 0.76)
Tilford et al. (2005) [56]	Children with spina bifida	A Trend test across the case group revealed that lesion level had a significant effect (at *p* = 0.01 level) on average utility score, with individuals with thoracic lesions scoring the lowest utility scores on average.	NA	NA	NA	NA
Usuba et al. (2014) [57]	Adolescents and adults with cerebral palsy	NA	NA	Average utility scores varied depending on PBM used; HUI3 derived utility scores were lower at baseline (0.29) and 8-year follow-up (0.29) than equivalent AQoL derived utility scores (0.35 and 0.32 respectively); statistical significance not reported.	NA	The ‘older adult’ group were more likely to report utility deterioration than the ‘younger adult’ group (HUI3: Relative risk [RR] = 1.19; 95% CI 0.66–2.15 / AQoL: RR *=* 3.17; 95% CI 1.12–9.00). A significant interaction was found between age group and time of survey using the AQoL (*p* = 0.002); AQoL derived utility improved over time in the ‘younger adult’ group but the opposite occurred in the ‘older adult’ group. The distribution of PBM dimension scores was stable across the 8-year follow-up, except for the AQoL social relationships, AQoL independent living and HUI3 ambulation dimensions, which all improved in the ‘younger adult’ group but deteriorated in ‘older adult’ group.
Vitale et al. (2001) [58]	Adolescents with orthopaedic problems (relevant condition: cerebral palsy)	There was found to be a significant difference (*p* > 0.05) between the mean utility scores of children with cerebral palsy (mean 0.92) and children with scoliosis with comorbidities (mean 0.725), but not children with idiopathic scoliosis (mean 0.889). Justification for these groupings was based on sample size, thus there is no explicit explanation as to why utility differences between these groups would be expected.	NA	NA	NA	NA
Wallander et al. (2009) [59]	Adults treated for CTEV in infancy	Male participants had significantly higher average utility score than the comparable norm group (*p* = 0.027). Male participants reported significantly less moderate/extreme problems with anxiety/depression than the comparable norm group (6.3% vs 21.2%; *p* = 0.007). Female participants had worse utility on average than a comparable norm group, but not significantly. Female participants reported significantly more moderate/extreme problems with mobility (45% vs 12.2%; *p* < 0.001) and usual activities (35% vs 12.5%; *p* = 0.006) than a comparable norm group.	NA	NA	NA	NA
Young et al. (2010) [22]	Adolescents and young adults with cerebral palsy	In both the ‘youth’ and ‘adult’ groups, utility scores deteriorated steadily as GMFCS level increased. Statistical significance not reported	Linear regression analysis revealed that GMFCS level in childhood was the most important influence on utility scores; responsible for 53.2% of variance in HUI3 utility outcomes (β = − 0.205; *p* < 0.001) and 45.2% of variance in AQoL utility outcomes (β = − 0.148; *p* < 0.001). The SRH was found to be moderately correlated with utility scores derived from the HUI3 (*r =* 0.41; *p* < 0.001) and AQoL (*r =* 0.41; *p* < 0.001).	Utility scores derived from HUI3 were on average higher than those derived from AQoL across all GMFCS levels; however strong correlation was found between the HUI3 and AQoL (*r =* 0.87; *p* < 0.001).	Proxy utility scores were generally lower (by 0.16) when adjusted for cognition, general health and CP severity. Statistical significance not reported.	NA
Young et al. (2013) [21]	Adolescents and young adults with spina bifida	Utility scores derived from both HUI3 and AQoL varied in the same way according to lesion level, with thoracic lesions associated with lowest utility. Linear regression analysis revealed that the most important single factor contributing to utility outcomes was surgical lesion level; responsible for 40% of variance in HUI3 utility scores and 18% in AQoL utility scores. Both lesion level and age were important when combined, accounting for 48% variance in HUI3 utility scores and 22% variance in AQoL utility scores.	The SRH was found to be moderately/strongly correlated with utility scores derived from the HUI3 (*r =* − 0.45; *p* < 0.001) and AQoL (*r =* − 0.58; p < 0.001). The HAQ was also strongly correlated with utility scores derived from the HUI3 (*r =* 0.79; *p* < 0.001) and AQoL (*r =* 0.70; *p* < 0.001).	Utility scores derived from the AQoL were higher than from the HUI3 across all lesion level sub-groups; however the AQoL and HUI3 exhibited strong correlation (*r =* 0.73; *p* < 0.001).	Mean utility scores were slightly higher in the self-reported group (HUI3 mean + 0.04; AQoL mean + 0.03) however this was based on the comparison of youth and adult data and the regression models remained unchanged.	NA

### Cerebral palsy

#### Known-group analyses

Five studies reported known-group analyses in CP [22, 33, 51, 53, 58]. Petrou and Kupek [51] estimated that the adjusted HUI3 disutility of childhood CP from perfect health was − 0.72 (95% confidence interval [CI] -0.61 to − 0.85), and − 0.65 (95% CI − 0.54 to − 0.78) from childhood norms. Two studies found that as CP severity (i.e. gross motor function) increased, average utility scores (measured using HUI3 or AQoL) decreased in adolescents and young adults with CP [22, 53]; Rosenbaum et al. [53] found statistically significant differences in mean utility scores between most Gross Motor Function Classification System (GMFCS) levels (p < 0.01). One study demonstrated that the vision, pain and cognition dimensions of the HUI3 steadily declined as GMFCS level increased, however statistical significance was not reported [33]. Vitale et al. [58] found that adolescents with CP had significantly higher average EQ-5D utility scores (0.92) compared to adolescents with scoliosis (with comorbidities) (0.73; *p* > 0.05), although selection of these patient sub-groups was not explicitly justified and the version of the EQ-5D was not reported.

#### Convergent validity: comparing measures

Two studies reported that GMFCS level was correlated with worsening utility scores [22, 53]. Young et al. [22] found that GMFCS level in childhood was responsible for between 45% (AQoL: β = − 0.148; *p* < 0.001) and 53% (HUI3: β = − 0.205; *p* < 0.001) of variance in utility scores. Rosenbaum et al. [53] found a strong negative correlation between the HUI3 utility scores of adolescents with CP and their GMFCS level (*r* = − 0.81). In terms of individual PBM dimensions, results from four studies were varied [33, 35, 39, 49]; Kennes et al. [39] found that the dimension most associated with GMFCS level in children was ambulation (tau-b = 0.82; *p* < 0.01). Bartlett et al. [33] found that the HUI3 vision, pain and cognition dimensions generally worsened as GMFCS level increased in adolescents; however, there was no indication that these dimensions were determinants of motor capacity decline. Two studies [35, 49] reported a significant negative association between the HUI3 pain dimension and GMFCS level in children with CP, however both of these studies only examined the HUI3 pain dimension and not the full HUI3 system.

Three studies reported various levels of correlation between PBMs and other outcome measures in CP [22, 46, 53]. Young et al. [22] found moderate correlation between utility score and SRH (*r* = 0.41; *p* < 0.001 for both the HUI3 and AQoL). Two studies compared the Quality of Life Instrument for People with Developmental Disabilities (QOL Instrument) and the HUI3 [46, 53]; Rosenbaum et al. [53] reported that adolescents’ HUI3 utility scores explained between 3% (belonging) and 14% (being) of variance in QOL Instrument dimension scores, while Livingston and Rosenbaum [46] reported that the HUI3 and QOL Instrument shared up to 23% variance, thus the relationship between the measures was considered to be moderate at best.

Two studies reported on the relationship between different PBMs in CP [22, 57]. Although utility scores derived from the HUI3 and AQoL were strongly correlated (*r* = 0.87; *p* < 0.001) [22], HUI3 derived utility scores tended to be lower than AQoL derived utility scores [22, 57].

#### Convergent validity: comparing respondents

Four studies examined correlation between respondents in CP [22, 48–50]. Morrow et al. [48] reported moderate agreement between parents and doctors of children with CP for the HUI2/3 dimensions of sensation (63.6% agreement; Kappa 0.41), cognition (70% agreement; Kappa 0.56), self-care (100% agreement; Kappa 1.00) and ambulation (63.6% agreement; Kappa 0.46). Good correlation was also found between child self-reported HUI3 pain scores and equivalent parent proxy scores (Goodman and Kruskal’s y statistic = 0.57; *p* < 0.001) [49].

Perez Sousa et al. [50] reported a high level of disagreement between parents and children on the EQ-5D youth version (EQ-5D-Y); parents reported a lower frequency of problems on all EQ-5D-Y proxy dimensions, particularly fathers.

#### Responsiveness

Five studies allowed analysis of PBM responsiveness in CP [33, 35, 46, 55, 57]. Adolescents with a GMFCS level of V exhibited the largest decreases in HUI3 dimension levels over time, compared to adolescents with GMFCS levels of III and IV [33]. Christensen et al. [35] reported a significant association between physicians’ primary pain aetiology and change in HUI3 pain status (*p* = 0.001). Conversely, in two studies utility outcomes did not change significantly over time; HUI3 utility outcomes were found to be stable over a 1 year period for adolescents with CP (G = 0.91) [46], likewise utility outcomes (derived from HUI3 and AQoL) did not significantly change over an 8 year follow-up period [57]. Slaman et al. [55] utilised the SF-6D in a randomised controlled trial, but did not find a significant difference between the control and intervention groups at the end of the trial (*p* = 0.42).

### Spina bifida

#### Known-group analyses

Three studies reported known-group analyses in SB [51, 52, 56], two of which found that clinical factors had a significant impact on utility scores. Petrou and Kupek [51] estimated that the adjusted HUI3 disutility of childhood SB from perfect health was − 0.55 (95% CI − 0.40 to − 0.70), and − 0.48 (95% CI − 0.33 to − 0.63) from childhood norms. A statistically significant effect of SB diagnosis on HUI3 utility score (*p* < 0.001) was reported [52]; children diagnosed with myelomeningocele (mean utility score = 0.51) tended to have lower utility scores compared to children with closed dysraphism (mean utility score = 0.77). Tilford et al. [56] reported that lesion location in childhood SB had a significant impact on overall utility (*p* < 0.01); individuals with sacral lesions had the highest overall mean utility (0.61; ±0.26).

Two studies reported correlation between clinical factors and utility scores in SB [21, 38]. Anatomical myelomeningocele level was found to have a significant effect on the HUI utility scores of children with myelomeningocele and shunted hydrocephalus (mean HUI score = 0.03; 95% CI 0.01 to 0.05; *p* = 0.01) [38], with lower myelomeningocele level showing association with higher utility scores. A similar trend was reported by Young et al. [21], who found that the most important single factor contributing to utility outcomes in SB was surgical lesion level, which was responsible for between 18% (AQoL) and 40% (HUI3) of variance in utility scores.

#### Convergent validity: comparing measures

Two studies reported various levels of correlation between PBMs and other outcome measures in SB [21, 54]. Child self-reported HUI3 utility scores were not found to be highly correlated with VAS scores (*r* = 0.488) [54]. Likewise, the SRH was only moderately correlated with utility scores in SB (HUI3: *r* = − 0.45; *p* < 0.001 / AQoL: *r* = − 0.58; *p* < 0.001) [21]. Only the HAQ was found to be strongly correlated with utility score (HUI3: *r* = 0.79; *p* < 0.001 / AQoL: *r* = 0.70; *p* < 0.001) [21].

Young et al. [21] compared results from different PBMs in SB, and found that mean utility scores on the AQoL were lower than mean utility scores on the HUI3 for all sub-groups, despite strong correlation between these measures (*r* = 0.73; *p* < 0.001).

#### Convergent validity: comparing respondents

Sims-Williams et al. [54] reported that proxy and self-reported HUI3 utility scores were highly correlated for children with SB (*r* = 0.85; significance not reported). Young et al. [21] found that mean self-reported utility scores were slightly higher than equivalent proxy scores (HUI3 mean + 0.04; AQoL mean + 0.03) however respondent type was not influential in their regression analysis.

PBM responsiveness outcomes in SB were not found in the literature.

### Mixed patient groups and mobility impairments

This section includes results from studies which did not focus on specific conditions, and where sub-group data could not be examined separately. Relevant conditions/mobility impairments included muscular dystrophy, spinal muscular atrophy, CP, SB, orthopaedic lower limb deformities, artrogryposis multiple congenital, achondroplasia and hemiplegia.

#### Known-group analyses

Two studies reported known-group analyses in studies of mixed patient groups [11, 51]. Petrou and Kupek [51] found that children with muscular dystrophy or spinal muscular atrophy had a mean utility score of 0.39, equating to an adjusted disutility from perfect health of − 0.62 (95% CI − 0.471 to − 0.761), and an adjusted disutility from childhood norms of − 0.54 (95% CI − 0.400 to − 0.690). Burstrom et al. [11] reported that children with a functional disability (64% congenital; see Table 4 for patient characteristics) had significantly lower EQ-5D-Y dimension scores than the general population (*p* < 0.001).

#### Convergent validity: comparing outcomes

Moderate correlation was found between the individual dimensions of the EQ-5D-Y and the dimensions of other measures (KIDSCREEN-27 and KIDSCREEN-10) and the life satisfaction ladder (LSL), when completed by children with functional disabilities [11] . The EQ-5D-Y anxiety/depression dimension exhibited significant correlation with the KIDSCREEN-27 psychological well-being dimension (*r* = − 0.51; p = 0.001), the KIDSCREEN-27 physical well-being dimension (*r* = − 0.53; *p* = 0.001), and the LSL (*r* = − 0.54; *p* < 0.001) [11].

Large variance was observed between utility scores derived from different PBMs for young wheelchair users; mean utility scores ranged from 0.24 (EQ-5D-Y) to 0.53 (HUI2) for self-reporting children, and from 0.01 (EQ-5D-Y) to 0.49 (HUI2) for parent proxies [15].

#### Convergent validity: comparing respondents

A significant strong correlation was observed between dyads of young wheelchair users (84.6% children with CP, see Table 4 for patient characteristics) and parent proxies for a number of utility measures: EQ-5D-Y (*r* = 0.67; *p* = 0.026), HUI2 (*r* = 0.73; *p* = 0.005) and HUI3 (*r* = 0.84; *p* < 0.001) [15]. Using Bland-Altman plots [60], sufficient agreement was observed between dyads for the HUI2 (CL = 0.22) and HUI3 (CL = 0.22), but not the EQ-5D-Y (CL = 1.04). A significant respondent type effect was found for all measures, with child self-reported utility scores significantly higher for the EQ-5D-Y (*p* = 0.012), HUI2 (*p* = 0.021) and HUI3 (*p* = 0.009) than equivalent proxy measures [15].

PBM responsiveness outcomes were not found in the literature for this patient group.

### Childhood hydrocephalus (mixed aetiology)

#### Known-group analyses

Lindquist et al. [45] found that adults with a history of hydrocephalus (with or without neuro-impairment) had significantly lower 15D dimension scores, compared to a control group, in the dimensions of vision (*p* = 0.001), eating (*p* = 0.000), usual activities (*p* = 0.004) and mental function (*p* = 0.000).

#### Convergent validity: comparing measures

Four studies examined the relationship between the Hydrocephalus Outcome Questionnaire (HOQ) and PBMs [40–43], however only three of these studies performed relevant statistical analyses [40, 41, 43]. Kulkarni [41] reported strong correlation (0.81) and a strong linear relationship between HUI2 utility score and HOQ outcomes for children with hydrocephalus. Simple and complex linear regression models both accounted for a large proportion of HUI2 variability (adjusted R^2^ = 0.66 and 0.80 respectively). Similarly, a strong correlation was found between HUI2 utility score and the HOQ scores for overall health (*r* = 0.81), physical health (*r* = 0.88), social-emotional (*r* = 0.56) and cognitive (*r* = 0.57) [40]. Furthermore, a significant positive correlation was exhibited between self-reported scores on the child-completed version of the HOQ (cHOQ) and proxy-reported utility scores on the HUI3 (*r* = 0.60; *p* < 0.001) [43].

PBM responsiveness outcomes in childhood hydrocephalus were not found in the literature.

### Other conditions and mobility impairments

Only known-group analyses were reported for the following conditions associated with mobility impairment:

#### Muscular dystrophy

Landfeldt et al. [44] reported that ambulatory status and age were significantly associated with HUI utility scores in muscular dystrophy (HUI version not stated; p < 0.001); young ambulators (5–7 years old) had the highest utility scores on average (0.75), whilst older non-ambulators (≥16 years old) had the lowest utility scores on average (0.15).

#### Spinal muscular atrophy

Lopez-Bastida et al. [47] reported that children with Type II spinal muscular atrophy tended to have lower mean proxy utility scores (− 0.01; ±0.35) than the combined average for all forms of spinal muscular atrophy (0.16; ±0.44), however statistical analysis was not undertaken.

#### Morquio A syndrome

One study found that for both adults and children with Morquio A syndrome, wheelchair use was significantly associated with lower utility scores [37]. Significant differences were reported in the adult group between non-wheelchair users and occasional wheelchair users (*p* = 0.0115) and between occasional wheelchair users and full-time wheelchair users (*p* = 0.0007). In the child group significant differences were reported between non-wheelchair users and full-time wheelchair users (*p* = 0.0018) and occasional wheelchair users and full-time wheelchair users (*p* = 0.0007).

#### Congenital clubfoot

Wallander et al. [59] found that male adult patients with congenital clubfoot (CCF) had significantly better overall utility scores than the male norm group (p = 0.027). The female CCF group had a lower average utility score than the norm group, but not significantly (significance level not reported).

#### Microcephaly

Petrou and Kupek [51] found that children with microcephaly had a mean utility score of 0.14, equating to an adjusted disutility from perfect health of − 0.82 (95% CI − 0.67 to − 0.97), and an adjusted disutility from childhood norms of − 0.75 (95% CI − 0.60 to − 0.90).

## Discussion

The results from this systematic review demonstrate that PBMs have been used in a relatively small number of studies relating to congenital mobility impairments. In conditions such as CP and SB, increased clinical severity appears to be associated with decreased utility. This is particularly evident using the HUI3, which was also the most commonly used PBM found in this review. In particular, there appears to be a relationship between utility outcomes and GMFCS level in CP, and clinical factors such as lesion level in SB. In case-control studies, utility outcomes tended to be significantly lower in the case groups, although it is worth noting that in one study the male case-group had significantly higher utility outcomes compared to the control [59].

In order to demonstrate sufficient applicability and sensitivity in a specific disease or disability, association between PBMs and validated clinical/condition-specific outcomes is of key importance. In this respect existing PBMs show weakness in various conditions associated with congenital mobility impairments; exhibiting generally limited correlation with measures such as the QOL Instrument, VAS, SRH, KIDSCREEN-27, KIDSCREEN-10 and LSL. Only the GMFCS and HOQ/cHOQ appeared to be well correlated with PBMs across a number of studies, although it is important to note that GMFCS is a classification system of gross motor function and not an outcome measure.

The results from this systematic review highlight important considerations for the use of PBMs in health states associated with congenital mobility impairment. It is first of note that the use of PBMs has been dominated by studies in CP, followed by SB. This is somewhat unsurprising given that these are two of the most prevalent congenital disabilities which affect mobility. Secondly, it is important to acknowledge that only two studies focussed on adults alone, and that both of these studies were measuring utility outcomes in adults following conditions experienced in childhood [45, 59]. This focus on children and adolescents is again unsurprising, as many congenital conditions can be life-limiting, thus examining health outcomes at a young age becomes particularly important. However, this also shows a lack of focus on the health outcomes of adults who have life-long experience of mobility impairment, and the potential ways in which their health outcomes could change over time or be improved.

There is some evidence to demonstrate that different PBMs vary in their estimation of utility outcomes in states of impaired mobility. For instance, Bray et al. [15] found large variation between the EQ-5D-Y and HUI2/3 utility scores for wheelchair users. Likewise, Usuba et al. [57] and Young et al. [21, 22] found that utility outcomes were generally higher when derived from the AQoL than the HUI3 for individuals with CP, but vice versa for individuals with SB. This is despite the strong correlation between the AQoL and HUI3 in these populations [21, 22]. Unfortunately, statistical differences between these measures were not reported, but it is still important to consider the implications that these differences could have on subsequent QALY outcomes. For instance, if one PBM were to produce significantly higher utility scores than another, this would mean significantly different estimates of cost per QALYs and thus estimates of cost-effectiveness. At present there is limited evidence to enable health economists and researchers to choose between these different PBMs for use in congenital mobility impairments.

Despite documented limitations [61], QALYs have become increasingly influential in health policy as a means to determine the cost-effectiveness of new treatments and services, and therefore guide healthcare funding and prioritisation decisions. It is therefore imperative that the PBMs used to develop QALYs are accurate. Otherwise, the cost-effectiveness of certain interventions in certain patient groups could be underestimated. This in turn could impact funding and prioritisation decisions. In the context of congenital mobility impairment, this could impact the provision of AMT and other mobility-enhancing interventions, and subsequently impact patient outcomes. This is particularly important considering the ongoing issues of unmet need in assistive technology provision. The WHO Priority Assistive Products List (APL) was launched in 2016 to help tackle unmet need [62]. The APL contains 50 priority assistive technology products, and was produced through consultation with users, experts and other key stakeholders. Appropriate PBM data, and subsequent estimates of cost-effectiveness, could help to inform the APL and ensure that the most effective and cost-effective AMTs are prioritised.

Considering that the majority of evidence found in this review related to children, the relationship between self-reported and proxy utility outcomes is particularly significant. The results from various studies in this review demonstrate that proxy-reporting of utility is consistently different to that of self-reporters, including significant respondent-type effects and limited agreement between respondents. Interestingly, proxy respondents were found to both underestimate and overestimate utility outcomes compared to self-reporters. It is therefore important to prioritise outcome measurement from the patient, although this can be challenging in populations who may lack capacity, such as young children and individuals with cognitive impairments.

### Methodological implications

Due to the underlying trade-off between quantity and quality of life in the calculation of utility values and subsequent QALYs, there is a tendency for lower value to be placed on extending the length of life of people with long-term disabilities [10, 63], as their quality of life is routinely considered to be worse than that of an able-bodied person. Thus, when using the QALY framework to assess the outcomes of individuals with disabilities, it is difficult to achieve substantially higher quality of life when compared to individuals without disabilities, raising concerns about bias [10]. To some extent these issues could be a result of using generic PBMs to value disabled health states.

One of the underlying issues of using PBMs in disability is that the definition of HRQoL differs profoundly between people with disabilities and the general public [64]. When asked to define HRQoL, young wheelchair users focus on a number of concepts not explicitly measured using generic PBMs, such as ability to adapt, achievement and independence [17]. The experience of disability also affects HRQoL perceptions. Mechanisms of adaptation, coping and adjustment can help individuals with disabilities to experience diminishing effects to their HRQoL over time. These processes are also influenced by the onset of disability, as individuals with congenital disabilities demonstrate better adaptation than individuals with acquired disabilities [2]. The evaluation of states of disability by non-disabled individuals may therefore cause such states to have an exaggerated perceived impact on HRQoL and health status [65], particularly with regards to congenital disability.

When assessing the desirability of hypothetical health states, individuals focus on the transition from their own health state to the hypothetical health state, thus general public beliefs about the impact of disability do not always reflect the lived experience [66, 67]. Focus on personal transition means that processes such as adaptation are not accounted for, causing a discrepancy in how states of disability impact HRQoL [9].

An alternative approach is to use more sensitive condition-specific measures to model utility values on generic PBMs. Although this approach is advocated by NICE [68] there are serious concerns about the validity of modelled utility values [69], as it cannot be assumed that modelled utility values are representative of directly measured utility values [70]. Using modelled utility data to guide funding and resource allocation decisions is therefore controversial. For instance, Sidovar et al. [71] mapped the 12-Item Multiple Sclerosis Walking Scale (MSWS-12) onto the EQ-5D-3 L. While prediction estimates were relatively precise for patients with moderately impaired mobility, they were significantly less accurate for individuals with severe impairments.

Neilson et al. [72] suggest supplementing PBMs with additional questions relating to functional activities which have a large impact on overall quality of life, such as ‘sitting’ for AMT users. For instance, Persson et al. [73] added complimentary mobility and social relationship items to the EQ-5D-3 L to increase sensitivity in disabled populations. However, this approach assumes that supplemental questions can be mapped on to the health state preference values of existing measures without impacting accuracy.

### Limitations and challenges

Defining the term congenital mobility impairment was one of the key challenges of designing this systematic review. An NHS posture and mobility service manager was consulted to help construct the search terms, and a number of preliminary searches were carried out to test search terms for both sensitivity and scope. A multitude of conditions and disabilities can affect mobility from birth or early infancy, thus we attempted to cover a wide variety of these in our search terms, but accept that there are likely to be conditions and disabilities which were missed. Given the search results, we are confident that we have captured the vast majority of relevant studies relating to at least the most common forms of congenital mobility impairment. One key issue was considering whether to include studies relating to hydrocephalus in this review. Although hydrocephalus is not always associated with mobility impairment, it can cause movement issues and is commonly related to relevant conditions such as CP and SB. We therefore chose to include studies related to childhood hydrocephalus.

We chose specifically to focus on congenital mobility impairment due to the significant differences in life satisfaction, self-identity and self-efficacy experienced by people with congenital disabilities compared to people with acquired disabilities [2]. Further research could compare PBM performance in congenital and acquired mobility impairments. Previous reviews have reported mixed results regarding the validity and responsiveness of existing PBMs in the assessment of health states associated with acquired mobility impairments, such as rheumatoid arthritis [24] and multiple sclerosis [26].

## Conclusion

To our knowledge, this is the first systematic review of the use of PBMs in congenital mobility impairment. Evidence suggests that existing generic PBMs exhibit important issues relating to validity and responsiveness, and thus care must be taken when selecting a PBM as an outcome measure in this context. Condition or disability specific approaches to utility measurement, such as the mobility and quality of life (MobQoL) outcome measure [74], could improve the sensitivity and applicability of utility measurement in this context.

## Data Availability

As this is a systematic review, there was no primary data generated as part of this research. All of the data utilised in this review is available from the cited literature, and has been summarised in Tables 4, 5 and 6.

## Abbreviations

AMT: Assistive mobility technologies
APL: WHO Priority Assistive Products List
APPT: Adolescent Pediatric Pain Tool
AQoL: Assessment of Quality of Life
BPI-SF: Brief Pain Inventory Short Form
CCF: Congenital clubfoot
cHOQ: Child-completed version of the Hydrocephalus Outcome Questionnaire
CI: Confidence interval
CP: Cerebral palsy
CRD: Centre for Reviews and Dissemination
CTEV: Congenital talipes equinus varus
DARE: Database of Abstracts of Reviews of Effects
DCGM-37: DISABKIDS Chronic Generic Module
DSM: DISABKIDS Smiley Measure
EQ-5D: EuroQoL Five-Dimension
EQ-5D-Y: EuroQoL Five-Dimension - Youth version
EQ-5D-3 L: EuroQoL Five-Dimension - Three Level version
EQ-5D-5 L: EuroQoL Five-Dimension - Five Level version
GMFCS: Gross Motor Function Classification System
GMFM-66: Gross Motor Function Measure 66 Items
HAQ: Health Assessment Questionnaire
HOQ: Hydrocephalus Outcome Questionnaire
HUI: Health Utilities Index
HRQoL: Health-related quality of life
HTA: Health Technology Assessment
LSL: Life Satisfaction Ladder
MeSH: Medical Subject Headings
MSWS-12: 12-Item Multiple Sclerosis Walking Scale
NHS: National Health Service
NHS EED: NHS Economic Evaluation Database
NICE: National Institute for Health and Care Excellence
PBM: Preference-based measure
PedsQL: Pediatric Quality of Life Inventory
QALY: Quality-adjusted life year
QOL Instrument: Quality of Life Instrument for People with Developmental Disabilities
QWB: Quality of Well-Being scale
RR: Relative risk
SAROMM: Spinal Alignment and Range of Motion Measure
SB: Spina bifida
SD: Standard deviation
SDQ: Strength and Difficulties Questionnaire
SF-36: 36-Item Short Form Health Survey
SF-6D: Short-Form Six-Dimension
SRH: Self-reported health
UN: United Nations
VAS: Visual analogue scale
WeeFIM: Functional Independence Measure for Children
WHO: World Health Organization
WRAT: Wide Range Achievement Test
